# Heiner syndrome and correlation with food allergy**:** case report

**DOI:** 10.3389/falgy.2025.1700662

**Published:** 2025-11-20

**Authors:** Paula Popovici, Răzvan M. Popovici, Maria-Alessandra Iuga, Elena Țarcă, Dana-Teodora Anton-Păduraru, Bogdan A. Stana, Lăcrămioara Ionela Butnariu, Radu Adrian Crișan-Dabija, Catalina Lunca, Alina Mariela Murgu

**Affiliations:** 1 Faculty of Medicine, “Grigore T. Popa” University of Medicine and Pharmacy, Iasi, Romania; 2 Pediatric Pneumology Department, St. Mary Children’s Emergency Hospital, Iasi, Romania; 3 Department of Obstetrics and Gynecology, Clinic Hospital of Obstetrics and Gynecology “Cuza Voda”, Iasi, Romania; 4 Pediatric Surgery Department, St. Mary Children’s Emergency Hospital, Iasi, Romania; 5 Third Pediatric Department, St. Mary Children’s Emergency Hospital, Iasi, Romania; 6 Department of Medical Genetics, Faculty of Medicine, “Grigore T. Popa” University of Medicine and Pharmacy, Iasi, Romania; 7 Pulmonology Department, Clinical Hospital of Pulmonary Diseases, Iasi, Romania; 8 Microbiology Department, St. Mary Children’s Emergency Hospital, Iasi, Romania

**Keywords:** Heiner syndrome, cow’s milk protein allergy, hemoptysis, food allergy, case report

## Abstract

Heiner syndrome (HS), although rare in pediatric practice, can cause cough, wheezing, and only in rare cases hemoptysis. HS should be considered in any child presenting these respiratory symptoms and radiological evidence of alveolar infiltrates, especially when accompanied by signs of food allergy—most notably cow's milk protein allergy, although other foods may also be involved. The use of probiotics should be evaluated with caution in these children. We report a case of a 1-year and 6-month-old female patient who presented at the Pneumology Clinic with a 1-day history of blood-tinged sputum. Although she had a known history of cow's milk protein allergy and well-controlled asthma, the occurrence of hemoptysis, even in small quantities, raised concerns for the family. A second episode of hemoptysis appeared when she consumed egg and probiotics. After correlating the patient's medical history with clinical, laboratory, and imaging findings, a diagnosis of HS was established. The patient received emergency treatment followed by bronchodilators, corticosteroid therapy, and a restricted diet for cow's milk protein and egg. The patient's condition improved immediately after treatment and remained stable at the 5-month follow-up. The differential diagnosis with idiopathic pulmonary hemosiderosis must not be overlooked, due to its more severe clinical course and higher risk of complications.

## 1 Introduction

Heiner syndrome is a rare type of hypersensitivity pulmonary disease with unknown global prevalence. It is mostly caused by cow's milk allergy, although eggs, wheat, and peanuts have been implicated in a few cases reported in the literature (1). Heiner syndrome manifests itself through recurrent episodes of pneumonia, but hemoptysis has rarely been reported in these patients (2). The authors present a case of Heiner syndrome in a pediatric patient who manifested hemoptysis after ingestion of mashed potatoes containing accidentally egg. This food-related hypersensitivity manifestation in children is very rare (2). The present manuscript describes the diagnostic challenges of Heiner syndrome and the particularities of this rare case involving multiple food allergies (cow's milk and egg whites) and food sensitizations (peanuts).

## 2 Case description

We present the case of an 18-month-old female infant admitted on an emergency basis to the Pneumology Department of “Sf. Maria” Children's Hospital in Iasi, Romania, for persistent cough, expiratory wheezing, dyspnea, and minor hemoptysis characterized by blood-tinged sputum. The onset of symptoms occurred 4 days prior to admission, presenting as cough and dyspnea in an afebrile context. The patient's parents initiated inhaled corticosteroid therapy (fluticasone 50 µg) in combination with a short-acting beta-2 adrenergic agonist (inhaler salbutamol 100 µg), with no significant clinical improvement. The emergence of hemoptysis raised parental concern and led to the presentation to the emergency department. The hereditary background is significant for atopy, with a maternal history of allergic rhinitis and a paternal history of bronchial asthma. Regarding the physiological personal history: she is the first-born child, delivered via cesarean section at 38 weeks of gestation, with a birth weight of 3,290 g and no signs of perinatal distress. She was vaccinated according to the national immunization schedule [anti-hepatitis and Bacillus Calmette–Guérin (BCG)] and exclusively breastfed until the age of 5 months. During the first 5 months of life, the patient developed mild atopic dermatitis [SCORing Atopic Dermatitis (SCORAD) = 24], for which local treatment and skin hydration were recommended, without the need for topical corticosteroid therapy. After the age of 5 months, with the introduction of dairy-based products, the patient’s atopic dermatitis worsened, progressing to a moderate-to-severe form (SCORAD = 40). This was accompanied by growth stagnation and a gradual onset of grade I protein–calorie malnutrition (nutritional index = 0.88). The patient also experienced her first episode of afebrile dyspnea associated with wheezing, which was interpreted as part of a bronchiolitis episode.

At that time, initial laboratory investigations revealed mild hypochromic microcytic anemia (Hb, 11.4 g/dL), marked eosinophilia (990/mm³), no evidence of inflammatory syndrome, normal coagulation profile, and elevated total IgE (104.3 IU/mL).Nasal eosinophils were absent, and polymerase chain reaction (PCR) tests for respiratory syncytial virus (RSV) and *Mycoplasma pneumoniae* were negative. Milk-specific IgE testing revealed a sensitization corresponding to class 5 (60 Ku/L). Additionally, sensitization was identified to casein, 16 Ku/L (class 3); egg white,0.36 Ku/L (class 1); dog epithelium, 0.62 Ku/L (class 1); and peanuts, 1.1 Ku/L (class 2). Based on these findings, the patient was diagnosed with cow's milk protein allergy (CMPA), and it was recommended that she receive a hydrolyzed protein formula and strictly avoid cow's milk and all dairy products.

Subsequently, over the following 12 months, the child experienced two additional episodes of respiratory insufficiency associated with wheezing and dyspnea, which responded rapidly and favorably to short-acting inhaled bronchodilator therapy (salbutamol 100 µg, four times daily) for 3 days. Based on this clinical course, a diagnosis of bronchial asthma was established, and maintenance therapy with fluticasone propionate (50 µg twice daily) was initiated. The parents administered both the inhaled corticosteroid and salbutamol inhaler at home prior to the patient's arrival at the emergency room.

At the current admission, the child presents a compromised general condition. She is afebrile and exhibits poor growth (nutritional index = 0.83). The skin is pale, with erythema of the cheeks, xerotic skin with fine scaling, mild pharyngeal congestion, dyspnea with tachypnea, and prolonged expiration. On auscultation, bilateral wheezing and sibilant rales are noted. Peripheral oxygen saturation shows normal values. The patient is hemodynamically stable, with normal stools and no other digestive symptoms.

**Figure 1:**
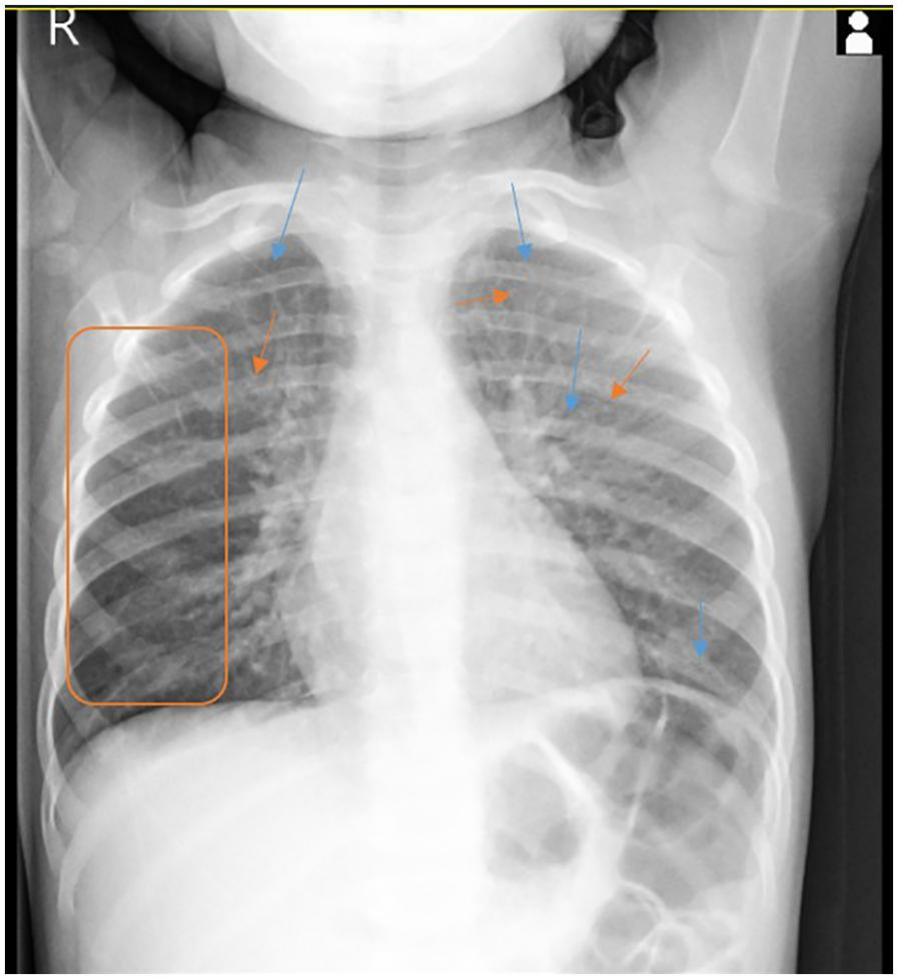
Posteroanterior chest x-ray shows a marked interstitial infiltration with a reticulonodular pattern in both lung fields.

Laboratory blood tests revealed mild anemia (Hb, 10.2 g/dL; Hct, 30%), with a hypochromic microcytic pattern, marked eosinophilia (1,400/mm³), and elevated total IgE (114 IU/mL). There were no signs of systemic inflammation, and liver and kidney function tests were within normal limits. Nasal secretions revealed eosinophils (qualitative test only). *Staphylococcus aureus* was isolated from the hypopharyngeal aspirate, while nasal and pharyngeal swabs were negative. The fecal occult blood test was also negative. Chest x-ray (performed for the first time) showed bilateral infiltration with a reticulonodular pattern (Figure 1). During hospitalization, the patient received initial treatment with systemic corticosteroids (hydrocortisone hemisuccinate 10 mg/kg/day), short-acting bronchodilator (salbutamol 100 µg, four times daily), hemostatic agents (etamsylate), and antibiotic therapy (cefuroxime 50 mg/kg/day).

**Figure 2:**
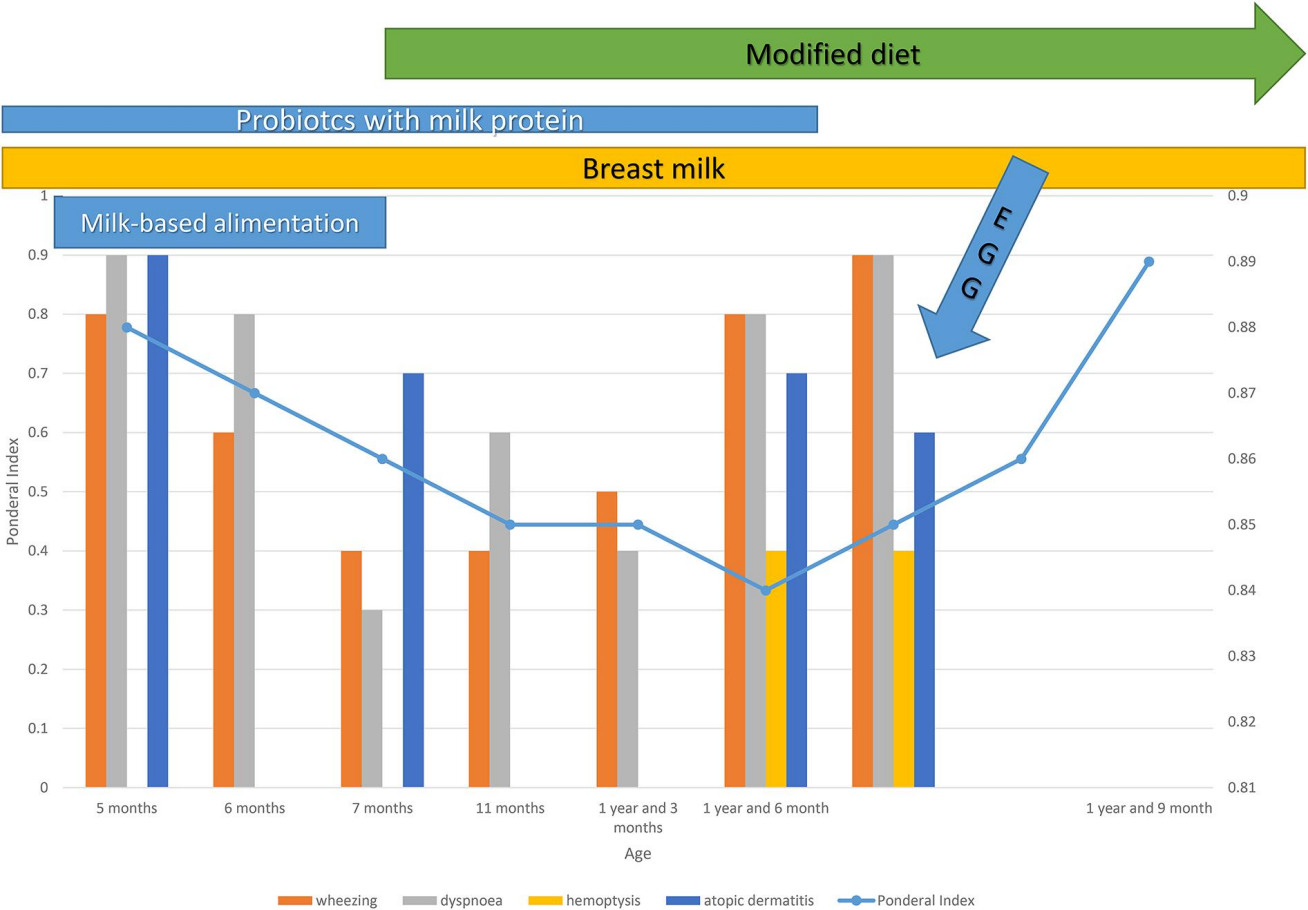
Long-term progression of allergic diseases over time.

**Figure 3:**
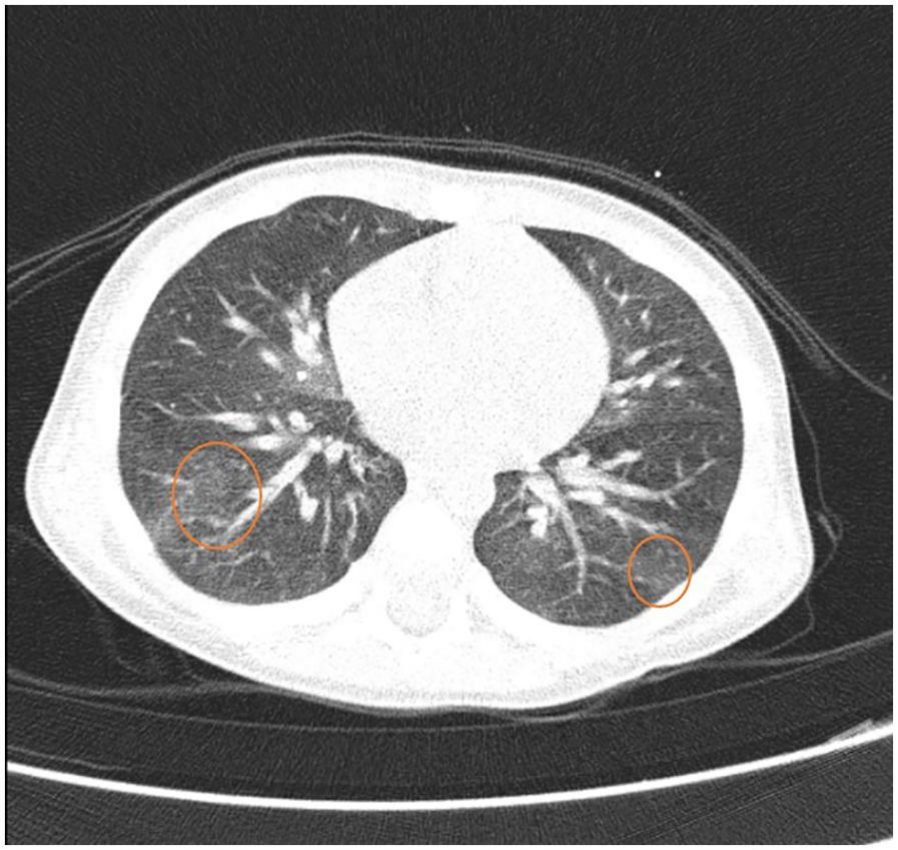
Chest CT scan shows pulmonary areas with normal attenuation alternating with a few small zones of slightly increased density, presenting a “ground-glass” appearance—suggestive of alveolitis, possibly of allergic origin.

On the 7th day of hospitalization, the patient develops an allergic reaction characterized by perioral erythema and edema, a generalized pruritic maculopapular erythematous rash, tachypnea, a repetitive episode of blood-tinged sputum, bilateral wheezing, sibilant rales, intercostal retractions, and mild hypoxemia (peripheral oxygen saturation of 95%). These symptoms occurred approximately 5 min after ingestion of approximately 100 g of homemade mashed potatoes that accidentally contained eggs. Apparently, no milk or dairy products were used in preparation, and all dishes had been thoroughly washed beforehand. The dynamics of clinical signs and symptoms are shown in Figure 2. Treatment with corticosteroids (hydrocortisone hemisuccinate 10 mg/kg/day), bronchodilators (salbutamol 100 µg, four times daily), and antihistamines (desloratadine 2.5 mg/day) was initiated, resulting in a rapid resolution of cutaneous and respiratory symptoms. Due to the persistence of intermittent wheezing, sudden-onset dyspnea, recurrence of hemoptysis, and more pronounced sibilant rales on the right hemithorax, a non-contrast chest CT scan was performed, with results suggestive of alveolitis (Figure 3).

At this point, a differential diagnosis between Heiner syndrome and idiopathic pulmonary hemosiderosis was considered due to the association of the clinical triad: anemia, hemoptysis, and diffuse alveolar pulmonary infiltrates. Bronchoalveolar lavage revealed hemosiderin-laden macrophages. Repeat allergy testing confirmed the presence of milk-specific IgE at similar levels: milk-specific IgE, 60 Ku/L (class 5), and casein-specific IgE, 16 Ku/L (class 3), with an increase of egg white-specific IgE, 2.2 Ku/L (class 2).

**Figure 4:**
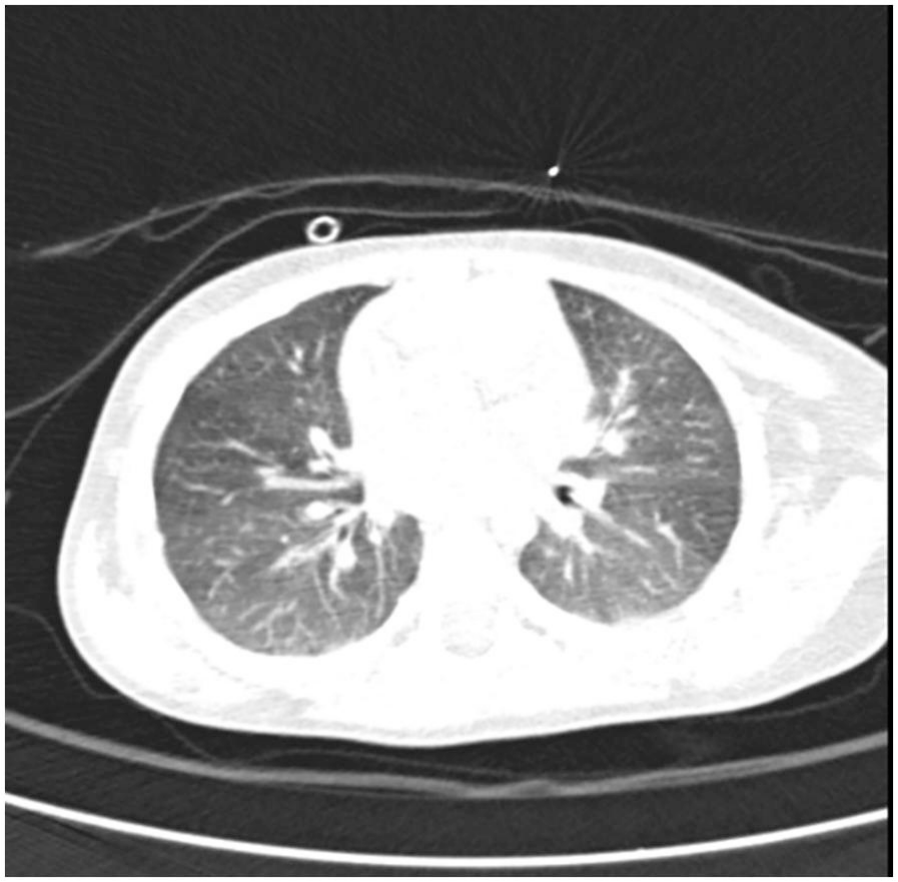
Follow-up chest CT scan revealing pulmonary areas with normal attenuation, without any focal lesions.

The patient is discharged in good general condition, with recommendations to continue inhaled corticosteroid therapy (fluticasone 50 µg twice daily) and maintain a strict elimination diet for milk proteins and egg-containing products. During the 3 months of follow-up, clinical evolution was favorable, with slight improvement in weight status (IP: 0.89), normal pulmonary auscultation, and no other pathological symptoms noted. Laboratory findings reveal persistent eosinophilia [730/mm³ (9.9%)] with no evidence of inflammatory syndrome. Fecal occult blood testing remained negative. Additionally, an improvement in anemia was observed, with a hemoglobin level of 11.5 g/dL. Considering the previously observed alveolitis-like changes on the native chest CT (occurring in the context of exposure to egg) and their resolution on the current CT with normal attenuation, without any focal lesions (Figure 4), a diagnosis of Heiner syndrome was confirmed. Furthermore, the presence of elevated milk-specific IgE levels and the favorable clinical response to a restrictive diet for egg and milk reinforced the diagnosis of Heiner syndrome.

## 3 Discussion

HS is a rare non-IgE-mediated hypersensitivity syndrome caused by cow's milk, characterized by chronic respiratory symptoms with chest x-ray infiltrates, and the resolution of signs and symptoms after a restricted diet. Although the disease was described in 1960 by Heiner et al. (3) in the USA, only a few cases have been reported to date. Hemoptysis is an uncommon manifestation; according to the literature, only 61 cases were reported until 2021, of which only 9 involved hemoptysis (14.75%) (4), with a few isolated cases reported later (5, 6).

The only meta-analysis of children with Heiner syndrome, conducted by Arasi et al. (4), included the first study published by Heiner et al. in 1960, which described seven children aged between 1 and 17 months with chronic cough, recurrent wheezing, intermittent fever, and hemoptysis in four of them (7). All children in the group exhibited recurrent respiratory symptoms, infiltrates on chest radiograph, and positive tests for allergy to cow's milk proteins (specific IgE and/or skin prick test).

In 1962, Holland et al. (8) reported the results obtained on the largest group of children published to date: 22 children aged between 4 and 12 months, with respiratory symptoms present in 17 patients; hemoptysis was not reported. Most patients showed sensitization to cow's milk proteins and responded favorably to the exclusion diet.

Another review from the literature, conducted by Moissidis et al. (9) in the USA, reported eight children diagnosed between 4 and 29 months of life. Most of them presented gastrointestinal signs, and two had hemoptysis. Although gastrointestinal symptoms predominated in this group (7/8), all cases presented radiological images of pulmonary infiltrates.

In 2014, Yavuz et al. (10) communicated a case of a 3-year-old boy admitted to the emergency unit with respiratory distress and hemoptysis. As in our case, the boy was known to have a cow's milk protein allergy and was on a restricted diet. In 2019, Koc et al. (11) reported the case of a 6-month-old boy presenting at the emergency department with massive hemoptysis, hematemesis, and deep anemia. The infant was treated previously for recurrent bronchopneumonia four times. He had many lung opacities on chest x-rays and on computerized tomography and hemosiderin-laden macrophages in fluid examination from his stomach, confirming the diagnosis of HS. His clinical and radiological recovery was completed with treatment and a CM-free diet.

In 2020, Chhawchharia et al. (5) reported the case of a 9-month-old girl who, like in the case, presented intermittent hemoptysis associated with post-tussive vomiting with mucus and blood clots for the past month. The biological and radiological picture was similar to the case presented by us, showing moderate anemia and diffuse infiltrates on chest radiograph, with a bilateral ground-glass appearance. Bronchoalveolar lavage was positive for hemosiderin-laden macrophages. Another similarity to our case would be the fact that this child also presented IgE-mediated to cow's milk and egg, but also a rice allergy was confirmed with an skin prick test. Avoidance of all this from the diet led to clinical improvement. A single ingestion of rice caused hemoptysis, suggesting that other food allergies, mediated by IgE, apart from milk, can cause hemosiderosis; therefore, the triggers should not be limited to milk and dairy products. Establishing a link to the IgE-mediated mechanism can elucidate the diagnosis. The reactions involved in the immune pathogenesis of HS are Gell and Coombs type III and IV, causing alveolar vasculitis by a less understood mechanism, which involves an immune complex and a cell-mediated response to cow's milk proteins (12). It has been speculated that pathogenesis is related to a non-IgE-mediated allergy to other food proteins, apart from cow's milk, such as soy, egg, pork, wheat, rice, and peanuts (4–6). Other authors postulated that milk antigens might trigger an immune complex reaction resulting in multiorgan abnormalities; pulmonary and gastrointestinal signs and symptoms are frequently associated (9). The same conclusion could be suggested in our case, presented in the current article, but it is not a certainty. The skin and respiratory symptoms occurred 5 min after the accidental ingestion of egg. The signs suggestive of HS were objectified on the chest x-ray on the following day. Our patient had a mixed allergy to multiple foods: milk, eggs, and peanuts. However, there is no evidence for the involvement of milk-specific IgE in HS.

The most recent case was published in 2024 by Ikobah et al. (6), presenting a misdiagnosis of a bronchopneumonia in a 9-month-old male child with cough, fever, dyspnea, and wheezing. The persistence of symptomatology despite an appropriate treatment and a positive maternal history of food allergy led to a review of the case. An allergy to cow's milk, eggs, and crayfish was revealed, and the elimination of these foods led to a complete resolution of the symptoms.

Global prevalence of HS is unknown, but it seems that approximately 5% of infants with cow's milk protein allergy have evidence of pulmonary infiltrates (13). Heiner syndrome manifests itself through recurrent episodes of pneumonia in which the following respiratory symptoms may be present: cough, wheezing, dyspnea, hemoptysis, rhinitis, otalgia, and adenoid tissue hypertrophy. Affected individuals typically present respiratory symptoms, but sometimes they may also have gastrointestinal symptoms such as abdominal pain, diarrhea, vomiting, hematemesis, and poor growth. All cases present radiological findings such as pulmonary hemosiderosis (9, 11, 14). This wide variability of clinical signs and their ability to mimic more common diseases makes diagnosis more difficult. Our patient was initially diagnosed with bronchial asthma due to a positive history of this disease. Because there is no specific test for Heiner syndrome, she was treated with inhalator corticosteroid, but her symptoms did not completely resolve. In the presence of the abovementioned clinical picture, several laboratory investigations can aid the diagnosis process. In HS, the pathogenic role of IgG to cow's milk is unknown, and their presence is neither sensitive nor pathognomonic. It has been stated that these antibodies may be found in 1% of healthy children and 4%–6% of children with chronic diseases such as celiac disease (4). Varying degrees of iron deficiency anemia and peripheral eosinophilia may also be found in some children. The patient in our study also presented eosinophilia and moderate anemia, with favorable evolution with a CM-restricted diet. In the majority of cases, high serum total IgE or circulating immune complexes may be present. Skin prick test and specific IgE levels to allergens may be negative in some patients with Heiner syndrome. Patchy and transient infiltrates can be revealed on pulmonary x-ray exams. In patients with pulmonary hemosiderosis, bronchoalveolar lavage, open pulmonary biopsy, or gastric lavage may reveal iron-laden macrophages (4, 15). The dietary exclusion of cow's milk causes a complete cessation of clinical and radiological findings in HS, and this fact is considered a major support for diagnosis. Reintroduction of cow's milk or an oral food challenge will cause a relapse of symptomatology and imagistic features (4, 11, 14, 16).

The diagnosis of HS was established in our patient due to clinical history and respiratory signs. The association of hemoptysis required a pulmonary CT scan that described lesions suggestive of pulmonary hemosiderosis. The remission of symptoms and of the radiological findings under the restrictive diet fully confirmed the diagnosis of Heiner syndrome. It is not possible to conclude that the consumption of egg products represented the trigger for the second episode of hemoptysis, but there are cases mentioned in the literature in which other foods can induce the specific symptomatology. For greater caution, the child required dietary elimination of cow's milk and eggs, especially because the allergy panel showed sensitization to egg white. In addition, after discharge, it was recommended to avoid peanuts, for which she also showed sensitization. The recommendation to avoid peanuts was made out of caution, even though a peanut allergy was not confirmed, only sensitization. The molecular test, component-resolved diagnostics (CRD), was not performed at that time for financial reasons, but we will take this into account during the next checkup.

The clinical improvement within days and radiological resolution of findings within weeks after elimination of cow's milk or other foods implicated is the main pillar of management for HS (4). The relapse of symptoms is usually caused by early reintroduction of cow's milk. In some cases, it has been reported that there has been a recovery without a food exclusion period. Within a few years, patients with HS may tolerate cow's milk by outgrowing hypersensitivity. After the elimination of cow's milk, there is no definite time frame for an oral food challenge to be tried. In acute attacks, immunomodulatory agents such as hydroxychloroquine or cyclophosphamide are usually used. In the rest of the cases that require additional therapy, bronchodilators, antihistamines, and systemic or inhaled steroids may be prescribed (15).

The patient in our study initially received systemic steroids and bronchodilators, as part of the management of bronchial asthma, with an improvement of symptoms. She continues to present intermittent wheezing, minor anemia, and poor growth, all these with strict CM-diet elimination. This is the reason why we believe that the ingestion of eggs might be the second trigger. The clinical evolution after elimination of eggs and cow's milk protein from the diet was favorable, with a complete resolution of symptoms and radiological findings. It is worth emphasizing that the little girl received a probiotic containing *Lactobacillus* until admission. We stopped the administration of probiotics because although it may modulate the immune response in allergies, there are some studies that show that the contamination of some preparations with milk proteins can have adverse effects for CMPA patients (17, 18). In addition, the use of probiotics and natural nutrition should be evaluated with caution, as they can cause allergic reactions up to anaphylaxis. Martín-Muñoz et al. (18) investigated in 2012 the safety of probiotics for subjects with food allergy. They studied 11 probiotics. Eight labels advertised lactose, lactic acid, or cow's milk, one label claimed to be milk-free, and two gave no information. Cow's milk proteins were detected in 10 cases, and three probiotics contained hen's egg white protein. The authors warned about the fact that probiotic compounds may contain hidden allergens and may not be safe for subjects with allergies to cow's milk or hen's egg. Multiple other studies support this hypothesis (17–19). There is insufficient data in the literature to associate the administration of probiotics with the risk of developing egg allergy, but in our case, the administration of the probiotic was stopped for safety reasons. Breastfeeding was recommended to be discontinued, considering the risk/benefit ratio at the age of 18 months.

## 4 Patient perspective

With appropriate treatment, the disease progressed favorably. They emphasized that understanding which foods and supplements may trigger a flare-up is crucial for the long-term management of a child with Heiner syndrome. It is necessary to perform a CRD test at the next allergist checkup to differentiate sensitization from peanut allergy and assess the risk of severe allergic reactions in the event of peanut ingestion. The authors also consider the risk of misinterpretation related to probiotic involvement in triggering the allergy.

## 5 Conclusions

Although rarely encountered in pediatric practice, Heiner syndrome should be suspected in young children with a history of food allergies, especially to cow's milk protein, but not only. Since there is no diagnostic score or other pathognomonic data, in the presence of suggestive chronic respiratory and/or digestive symptoms, as well as radiological findings, the exclusion diet is the key to excluding other pathologies with similar symptomatology. The clinical resolution after the restrictive diet usually confirms the diagnosis. Hemoptysis is not a frequent feature of the disease, but it usually causes an early presentation to a specialized emergency medical unit. Neglected hemoptysis will lead to severe anemia and potentially other serious complications.

## Data Availability

The original contributions presented in the study are included in the article/Supplementary Material; further inquiries can be directed to the corresponding author.
